# A Payer’s Perspective: A Comparison and Simulation of the Costs of Hemodialysis Versus Living Donor Kidney Transplant for Patients With End-Stage Renal Disease in Nigeria

**DOI:** 10.3389/ti.2022.10662

**Published:** 2022-07-20

**Authors:** Jacob J. Lang, Conner V. Lombardi, Iyore A. James, David B. Da Rocha-Afodu, Chimezie G. Okwuonu, Obi O. Ekwenna

**Affiliations:** ^1^ Department of Urology and Transplantation, University of Toledo College of Medicine and Life Sciences, Toledo, OH, United States; ^2^ Surgical Specialists of Charlotte, Matthews, NC, United States; ^3^ Department of Internal Medicine, Nephrology Unit, Federal Medical Centre, Umuahia, Nigeria

**Keywords:** dialysis, transplantation, policies, cost, access to transplantation, cost-comparison

Dear Editors,

Chronic kidney disease (CKD) carries substantial disease and economic burden in Nigeria. The prevalence of CKD in Nigeria has been estimated to range from 11.4% to 26% (1). Due to factors such as limited education on the early asymptomatic stages of CKD, poor screening practices, and limited nephrology care, many cases of CKD result in progression to end-stage renal disease (ESRD) (2). Living Donor Kidney Transplant (LDKT) is considered the gold standard treatment for patients with ESRD or stage five chronic kidney disease (CKD) (3). LDKT offers ESRD and CKD patients a significantly better quality of life and life expectancy than those receiving hemodialysis (HD) or other renal replacement therapies (4, 5). The feasibility of LDKT compared to other forms of RRT may differ on a country-by-country basis due to factors such as availability/accessibility of transplantation and HD centers, health insurance coverage for ESRD and CKD care, robustness of the donor organ procurement network, and government support (6).

In 2016, there were approximately 80 hemodialysis centers and five transplant centers in Nigeria (7). The outlook for patients with CKD and ESRD is poor, as there is no national insurance or health aid scheme (e.g., Medicare in the United States) to financially support these patients, leaving the financial burden of RTT fully upon patients and their families (8). Furthermore, there is no structured organ procurement and donation network in place in Nigeria, meaning that most kidney donors in Nigeria are usually genetically or emotionally related to the donor (6).

To date, data regarding the cost of LDKT and HD in Nigeria are limited. Through our experience with the Clarion Call transplant program across Western Nigeria since 2014, we aimed to address this gap. The aim of this study was to primarily quantify, compare, and conduct a simulation of costs of LDKT and HD in Nigeria from a payer’s perspective and secondarily inform future cost-effectiveness studies to guide health care decision-making of kidney disease management in the country.

Our analysis takes on the perspective of the ESKD patient payer in Nigeria. Data from the Clarion Call Transplant Program in Nigeria from 53 patients who underwent hemodialysis at centers across Nigeria from July 2014 to June 2020 and 20 patients who received a LDKT between June 2017 and May 2020 was used in this study. Cost estimates were determined through direct supplier pricing and patient utilization data, and confirmed by expert consultation *via* two transplant nephrologists, two transplant surgeons, and one transplant coordinator in Nigeria. All costs were reported in both 2020 USD and 2020 Nigerian Naira using the conversion rate in 2020. One year and yearly recurring costs of LDKT and HD were calculated to compare RRT modalities. HD costs were projected for both three sessions per week and two sessions per week to simulate a more feasible alternative, although three sessions per week is the standard of care in most resource-rich countries.

Costs and model inputs are shown in Table 1. We estimated one-year costs of US $23,096.32 for HD (three sessions per week) and $37,271.00 for LDKT. Yearly recurring costs were $22,394.32 for HD and $6,709.30 for LDKT. Costs of acute rejection for LDKT were $2,418.00 for antibody-mediated rejection and $1,170.00 for cell-mediated rejection. One-time costs dominated the one-year cost of LDKT at 82.0%, while alternatively 97.0% of one-year costs of HD were recurring costs. A discounted simulation (6% discount rate, as is recommended in resource-limited countries) of three-year costs when survival was assumed yielded a cost-savings for LDKT in comparison to HD of US $12,421.23 for three sessions per week, and $1,655.86 for two sessions per week.

**TABLE 1:** Costs of model inputs for hemodialysis, living donor kidney transplant, and acute rejection.

Treatment modality	Input	Description	Yearly cost (₦)	Yearly cost (USD)
Hemodialysis	Dialysis treatment (three sessions per week)	30,000 N per session	₦ 4,680,000	$12,168.00
	Dialysis treatment (two sessions per week)	30,000 N per session	₦ 3,120,000	$8,112.00
	Labs (pre dialysis)	Once per session	₦ 800,800	$2,082.08
	Labs (post dialysis)	Once per session	₦ 572,000	$1,487.20
	CVC placement	One time	₦ 150,000	$390.00
	AV fistula creation	One time	₦ 120,000	$312.00
	Epogen injection	Per protocol	₦ 1,560,000	$4,056.00
	Iron injection	Per protocol	₦ 312,000	$811.20
	Vitamin D	Per protocol	₦ 48,000	$124.80
	Phosphorus binders	Per protocol	₦ 80,000	$208.00
	B-Complex vitamin and folic acid	Per protocol	₦ 10,400	$27.04
	Nephrologist consultation	Per protocol	₦ 300,000	$780.00
	Nutritionist consultation	Once per year	₦ 250,000	$650.00
		First year total (three sessions per week)	₦ 8,883,200	$23,096.32
		Recurring costs (three sessions per week)	₦ 8,363,200	$22,394.32
		3 Year total (three sessions per week)	₦ 26,109,600	$67,884.96
		First year total (two sessions per week)	₦ 7,323,200	$19,040.32
		Recurring costs (two sessions per week)	₦ 7,053,200	$18,338.32
		3 Year total (two sessions per week)	₦ 21,429,600	$55,716.96
Living donor kidney transplant	Work up	One time	₦ 1,500,000	$3,900.00
	Transplant surgery	One time	₦ 10,000,000	$26,000.00
	Follow-up labs	One time	₦ 528,000	$1,372.80
	Tacrolimus (1 mg ×100 caps)	45,000 per unit	₦ 1,296,000	$3,369.60
	Cellcept (500 mg ×100 caps)	50,000 per unit	₦ 720,000	$1,872.00
	Prednisone (5 mg ×100 tabs)	10,000 per unit	₦ 36,500	$94.90
	Anti-viral	Valcyte	₦ 180,000	$468.00
	Anti-fungal	Fluconazole	₦ 36,500	$94.90
	Anti-bacterial	Bactrim	₦ 38,000	$98.80
		First year total	₦ 14,335,000	$37,271.00
		Recurring costs	₦ 2,580,500	$6,709.30
		3 Year total	₦ 19,496,000	$50,689.60
Acute rejection	Kidney biopsy	Per protocol	₦ 200,000	$520.00
	Anti-thymocyte globulin	Per protocol (cell-mediated)	₦ 250,000	$650.00
	IV immunoglobulin	Per protocol (antibody-mediated)	₦ 250,000	$650.00
	Plasmapheresis	Per protocol (antibody-mediated)	₦ 480,000	$1,248.00
		Total (cell-mediated)	₦ 450,000	$1,170.00
		Total (antibody-mediated)	₦ 930,000	$2,418.00

Note: 3 year totals are not discounted.

Costs of LDKT in Nigeria are higher than that of HD in the first year but are markedly decreased in subsequent years. The cost of HD and LDKT is primarily an out-of-pocket expense paid by patients with kidney failure in Nigeria. The maintenance cost of HD is three times more than the maintenance cost of immunosuppression post kidney transplantation. Our data demonstrates favorable long-term cost profile of LDKT vs. HD in Nigeria when cost is borne directly by patients. These potential cost-savings are in line with cost comparisons of the two RRT modalities in many settings globally and demonstrate the benefit of LDKT from a cost perspective.

This study considers no treatment as a non-viable option going forward. One study of a Nigeria HD center found median duration of treatment for those receiving HD in Nigeria to be as low as 1 week, with only 30% with continued dialysis after 3 months. Median survival for those on HD is abysmal, with median survival for females as low as 5 weeks and males 20 weeks (8). A larger, 6 years study of 1,167 patients from five HD centers across Northwestern Nigeria found rates of sustained dialysis past 90 days to be as low as 15.1% at one center, with only 41.7% of patients receiving more than three sessions in total, and only one patient referred for kidney transplant over the period from 2011 to 2017 (9). This study is limited by lack of data regarding access to RRT modalities or survival rates of either HD or LDKT, as well as the lack of data regarding complications including hospital visits, rejection rates, etc., and merits further study.

We found a favorable long-term cost of LDKT versus HD in patients with ESRD at our transplant program in Nigeria. Despite high up-front costs of LDKT, maintenance costs were demonstrated lower than that of HD. Cost data from this study can be used for further study of the comparative cost-effectiveness of these RRT modalities using survival data and outcomes to assess cost-effectiveness and help inform local policymakers with aim of increasing access to LDKT.

## Data Availability

The raw data supporting the conclusion of this article will be made available by the authors, without undue reservation.
